# Stability or versatility: transitions in fentanyl routes of administration

**DOI:** 10.1186/s12954-025-01373-y

**Published:** 2025-12-27

**Authors:** Jeff Ondocsin, Sarah G. Mars, Nicole Holm, Allison Schlosser, Jason Fessel, Amanda Cowan, Michael Duke, Daniel Ciccarone

**Affiliations:** 1 Family & Community Medicine Department of Medicine, University of California, San Francisco, San Francisco, USA; 2 Sociology and Anthropology Department, University of Nebraska Omaha, Omaha, USA; 3 Community Health Project Los Angeles, Los Angeles, USA; 4 Division of Health Equity and Society Department of Medicine, Center for Vulnerable Populations, University of California, San Francisco, San Francisco, USA

**Keywords:** Opioids, Fentanyl, Route of administration, Injection, Smoking, Overdose, US, Harm reduction services

## Abstract

**Background:**

Transitions in how people use drugs have long influenced drug use research and the policy landscape. In unregulated drug markets, route of administration (ROA) transitions from injecting opioids to non-injecting modes of use, sometimes called reverse transitions, are rare but have been documented historically, driven by local characteristics including social networks and drug market factors. National and local data find ROA transitions are on the rise in the US. We investigated patterns of ROA transitions, including personal and market-based motivations and perceived effects and health benefits.

**Methods:**

We examined trends in San Francisco’s drug market, including changes in substance use administration patterns and the forces motivating people who use drugs to adopt alternate or multimodal dosing strategies. We conducted 32 semi-structured qualitative interviews in 2022 using rapid assessment ethnography and observation to triangulate findings on ROA patterns and techniques among people using opioids.

**Results:**

We observed fluctuations in the relative availability of heroin and fentanyl which contributed to subsequent changes in ROA. Like other US locations, participants initiated fentanyl use as heroin availability diminished. Able to achieve comparable effects from fentanyl smoking relative to injection most participants indicated they had transitioned within the past several years, driven by venous access, overdose concerns and market characteristics. Transitions were graduated along a spectrum of stability, from frequent multimodal use to transitions in accord with recent patterns, notably featuring total cessation of injection. Others found combining smoking and injecting allowed greater flexibility, with smoking favored when in public, for convenience, considering sensitivity to non-injecting peers, or when seeking a milder experience enabling productivity or vigilance. Injecting was chosen for its faster effect, to avoid sharing drugs/equipment with others and especially for polysubstance combinations. A small subset of participants indicated that smoking was not a durable transition for them, necessitating a return to injection.

**Conclusions:**

The stability of ROA transitions and their health impacts remain in flux amidst widespread uptake of smoking fentanyl. Smoking supply provision and harm reduction organizations are facing notable headwinds but continue to be essential for providing people control over their health and embodied and social experiences.

## Introduction

The methods by which people use drugs has been a historic focus of substance use research, particularly regarding people who use drugs (PWUD). Administering drugs by injection presents the highest risk for health complications, whether from blood borne viruses including HIV or hepatitis C, overdose or bacterial infections like endocarditis [1]. People’s preferred ingestion routes are not static, however, but are subject to change over time [2]. These transitions, such as from non-injecting to injecting modes of use and the reverse, are therefore of considerable public health significance.

While some people use opioids for the first time by injection [3, 4] it remains rare. However those initiating by snorting or smoking often find themselves abandoning these methods over time in favor of injection, sometimes with reluctance and despite the heightened risks [5]. The development of opioid tolerance to the desired euphoric and analgesic effects leads those who use them by non-injection routes to seek out more efficient and intense modes of administration [2, 4, 6]. Drug bioavailability is typically highest when injected, and the twin demands of economy and efficiency often lead to injection initiation [7–9]. Route of administration (ROA) transitions are also substantially impacted by culture and by social network influences [10, 11].

Heroin use in particular is associated with relatively rapid initiation of injection as compared to other drugs [6, 9]. Of course, transitions to injecting are not universal or predetermined: early research among heroin smokers in the UK found that ‘chasing the dragon’ was a relatively stable ROA over time, and was not simply a brief phase before the inevitable uptake of injection [4, 12, 13]—although later research found injection use to be more constant than chasing [14]. The novel adoption of heroin smoking in the UK was enabled by the introduction of a smokeable form of heroin from Southwest Asia into a market where injected Southeast Asian heroin was the norm [15].

Much of the early work on ROA transitions focused on reducing HIV transmission among people who inject drugs. Studies from the 1980s and 1990s in the UK found a transition to injection from other modes most common among people using heroin [13]. Research from this period also introduced the concept of a ‘reverse transition’ where some users of heroin moved from injection to smoking or other ‘earlier’ forms of use [2, 4, 13] to reduce exposure to injection-related harms or to moderate their use [2, 13]. However, reverse transitions in the US have been seldom documented and limited in scope [16–19] but can be stable, long-term changes [16, 19] and similarly, research in Australia has found them to be relatively rare [20]. Heroin smoking has been seldom practiced in the US, likely influenced by the unsuitability for smoking of the hydrochloride salt form of heroin that was available in the US [21]. Particularly notable findings on ROA transitions from the UK indicate that primary ROAs were stable over time and that it was relatively uncommon for PWUDs to use multiple routes simultaneously [13]. Regional differences in mode of use and transition patterns may be pronounced, producing disparate outcomes across geographic areas. Studies carried out in the Netherlands in the 1980s and in Barcelona, Madrid and Sevilla in Spain during the 1990s confirmed earlier findings of a cohort effect encouraging uptake of non-injection modes of administration among new users of heroin and found that future changes in route of administration are often predicted by regional characteristics, i.e. local prevalence of smoking or injecting [22, 23].

The addition of fentanyl and related analogues to street opioids in the US over the last decade has been well described [24–26]. This process occurred first in the Eastern, Southern and Midwestern regions of the United States, with fentanyl not widely available in the Western region until approximately 2019 [27]. Once fentanyl became widely available in the West, it dramatically impacted overdose fatalities, which have doubled in California since December 2019 [28]. San Francisco’s overdose death rate has similarly skyrocketed, with a rate of 92.4 per 100,000 over the period from November 2022 to October 2023, twice the rate of the same time period four years earlier [29]. Death rates among minoritized racial groups in San Francisco continue to be substantially higher than the national average, particularly among African Americans [29]. Notably, fentanyl was involved in 78% of deaths over the 2022–2023 period [29], but it is often unclear from death data which ROA was associated with each death.

Fentanyl has been found to be stable during pyrolysis at temperatures up to 350°C [30], rendering it feasible for consumption through vaporization, or ‘smoking’, as it is commonly described [31]. Patterns of fentanyl smoking exhibit strongly regional trends, with smoking most prevalent in the US West [32], suggesting additional factors supporting this transition, e.g., historic patterns of substance use, drug supply characteristics, and differences in the timing of fentanyl’s entry into the drug supply. An observational cohort study in San Francisco carried out between 2018 and 2020 found drastic reductions in the median number of past-month opioid injections as days smoking fentanyl increased [33].While in other locations, fentanyl has been an unadvertised adulterant of the heroin supply [26], San Francisco quickly saw the emergence of a market where fentanyl was sold apart from heroin [31] which may have further accelerated smoking uptake.

Of possible comparative value to the current moment are heroin shortages that occurred in Australia in 2000–2001 and the UK in 2010–2011 [34, 35]. While these events were not also accompanied by the introduction of a potent new opioid into the street supply, they were characterized by ROA transitions, changes in consumption patterns and the substitution or addition of other drugs to the daily repertoires of PWUD [34, 35], similar to phenomenon that are currently occurring in San Francisco [31, 36, 37]. Earlier work published by this study team shows that the main motivators for transitions from injecting to smoking fentanyl were fear of overdose and loss of venous access [31]. This shift was facilitated by fentanyl’s low price, plentiful availability and high potency [31, 37].

In this paper we look in more detail at the varying patterns of transition between modes of fentanyl use, employing a broad scope to study transitions in drug use patterns, including the use of other opioids immediately prior to fentanyl uptake. The US-wide transition in drug use between opioids, where fentanyl was initially added to heroin before being sold explicitly, is well described in the literature, including by this study team [26, 38], but ROA transitions in the context of the current fentanyl market are less well understood. Drawing from our ongoing ethnographic work in naturalistic settings with people who use drugs, we examine novel ways in which research participants in San Francisco administer their fentanyl, particularly how they combine multiple ROAs based on considerations of effect, efficiency, polysubstance use, and social context. We then explore drug route of administration transitions in the context of these factors and contextualize the implications of these transitions for harm reduction interventions.

## Methods

This study uses a focused ethnographic approach to investigate current trends in polysubstance use and their influences on health and social outcomes among PWUD in San Francisco. Fieldwork occurred over three non-concurrent weeks in June, September, and November of 2022. Week 1 consisted of in-depth ethnographic observation and rapport building with the study population. Weeks 2 and 3 consisted of participant recruitment and interviewing in addition to ongoing observational activities. Participant recruitment took place in three neighborhoods in San Francisco—the Tenderloin, the Castro and Haight-Ashbury—through syringe service providers as well as at the Tenderloin Center, a temporary collaboration between the city and multiple service agencies to connect PWUD and the unhoused population in the Tenderloin/South of Market neighborhoods with health and social services. During the second half of 2022, the Tenderloin Center also housed a supervised consumption space. These locations were chosen for their proximity to the downtown neighborhoods where the open-air drug market is located, the different populations served by each site and previously existing relationships the research team had with these providers. Participant observation took place both at and outside of service provider locations, including the Tenderloin Center supervised consumption space, the neighborhoods where these services were located, and at participant’s homes or places where they used drugs. Observations occurred during service hours and outside of these hours, including overnight hours. To be included in the study, participants had to be 18 years old or older and currently using fentanyl and/or methamphetamine (past 30-day use) by any ROA, although this analysis focused only on individuals using fentanyl. Ethnographic team members approached individuals attending services, confirmed they met the inclusion criteria and attained verbal consent to participate. Some participants were introduced to interviewers by program staff who were informed of the inclusion criteria. Exclusion criteria included individuals who did not use fentanyl or methamphetamine or individuals who were incapable of providing verbal consent to participate in the study. Verbal consent was sought to protect participant confidentiality. Interviews were held in private rooms at two recruitment locations and were conducted outdoors or inside private vehicles at the other location.

We conducted semi-structured interviews with PWUD (*n* = 34), consisting of questions about substance use history, current polysubstance use habits, trends in ROA, drug markets, health outcomes including experience of overdose and other related topics. Two participants were excluded from this analysis due to solely using methamphetamine, reducing the sample to *n* = 32 (see Table 1). *N* = 30 participants indicated they had previously injected before transitioning to smoking. Interviews were audio-recorded and lasted approximately 45–60 min, for which participants were compensated $25. Additionally, some participants participated in video and photographic recording of drug equipment, preparation and consumption (*n* = 12) and were compensated an additional $25. The team wrote collaborative fieldnotes for each research day to record observations, informal conversations with participants, service providers or members of the public and other materials not captured by audio or video recording. Collecting these additional data helped to triangulate and contextualize findings explored in the interviews. Participants’ names have been pseudonymized to protect their confidentiality. We received human subjects approval from the University of California, San Francisco. Our research activities and the resulting data are protected by a Federal Certificate of Confidentiality issued by the National Institutes of Health.

**Table 1:** Participant demographic data (*n* = 32)

Gender		
	Male	16
	Female	13
	Transgender/non-binary	3
**Age (years)**		
	18–29	5
	30–39	13
	40–49	10
	50–59	4
Race/Ethnicity*		
	White	22
	Black/African American	3
	Hispanic/Latino	3
	Native American	5
	Multiracial	7
Current mode of use*		
	Injection	18
	Smoking	31
	Both smoking and injecting	10
**Injection history**		
	Ever injected a drug	30
	Ever injected fentanyl	21

*Participants were allowed to indicate multiple categories, resulting in counts greater than 32

### Analysis

Audio recordings of interviews were professionally transcribed and checked for accuracy by the authors. Transcriptions of audio from video recorded substance use sessions were completed by the authors. Analytic memos using the process outlined by Christopolous et al. [39] were prepared for each interview transcript, drawing initially on categories based on the interview guide before being expanded in an iterative process to include findings generated through close reading and weekly discussions among the authors (JO, SGM, NH, AS, JF). Analytic memos were written by one author (JO, SGM, NH, AS, JF, AC) before being reviewed and added to by a separate author. Afterwards, we used the analytic memos, fieldnotes, photographic and video recording data to develop several thematic memos based on notable findings identified in weekly discussion and focused on illuminating the key study questions (e.g., perceived concerns about injecting) to further condense and refine our findings. Photographs were organized by content (AS) and additional analytic memos were prepared for each video recording (AS). Authors met weekly to discuss findings and provide feedback on the analytic process. Differences in opinion and interpretations of findings often occur during data analysis. The authors reflected throughout the analysis on their positionality and any preconceived interpretations they may have brought to this process. Data analysis was conducted by several multidisciplinary researchers with unique life experiences, professional backgrounds, ages and mixed genders. When differences of interpretation arose, these were discussed among the authors until consensus was reached.

## Results

Participants ascribed both personal motivations as well as drug supply and market changes to explain why they transitioned from injecting opioids to smoking fentanyl. Transitions in this sense encompass both a transition in drug from one opioid (heroin) to another (fentanyl) as well as a transition in ROA from primary injection use to primary smoking use or mixed route of administration. While some described ROA transitions that correspond with reverse transition patterns seen with heroin use, others digressed from this pattern, describing their transitions as more fluid, multidirectional and multimodal than historical patterns. These differences in how participants expressed their own transition patterns from the transitions of previous eras raise important questions about the nature of ROA and substance use transitions and how they endure or dissipate over time. Participants described combining multiple modes of administration in their daily drug use, moving back and forth between routes of administration situationally and based on social, personal and economic contexts.

### Conditions facilitating change

When describing their own transitions, participants situated these changes in terms of changing tolerance to heroin and venous access, as well as a belief that smoked fentanyl presented a lower risk of overdose than injecting. They also framed the introduction of a more potent opioid as an opportunity to refrain from injecting, since smoking fentanyl was perceived as being at least as potent as injected heroin. As such, fentanyl’s arrival in San Francisco challenged the status quo, introducing a street drug that, through its production and distribution advantages, became far cheaper than heroin. Initially sold alongside heroin, fentanyl achieved primacy in the marketplace, while heroin reportedly declined in quality and became harder to obtain, particularly at prices that could compete with those of fentanyl [31, 37]. For those accustomed to heroin, this was a true paradigm shift, one that would result in significant disruptions to established patterns of opioid use. Lana, a cisgender woman in her 40s who had injected heroin before transitioning to smoking fentanyl (‘fetty’), explained:

*Lana: I got out of prison. I had no idea about fetty […] I was standing outside of my house*,* and I’m on [street] so I’m right in the shit. I’m standing out there*,* and I’m looking. Everybody is facedown*,* and I was like*,* “What the fuck*,* dude? The heroin’s hella good or what?” I didn’t get it. […] So*,* I was like*,* “I want to try this heroin.” […] And then that’s when everybody was like*,* “No*,* it’s fetty.”*

The transition to smoking did not immediately coincide with fentanyl’s introduction to San Francisco and participants described the challenges of the initial adjustment period to this new reality:

*Lana: People [were] learning how to do [fentanyl]. At first*,* people were going crazy with it. They were slamming [injecting] it*,* and they were fucking treating it like it was heroin. And it’s not heroin. Now*,* people understand what the fuck it is*,* and they’re doing it accordingly. […] Not everybody. People are always going to overdose with fentanyl. That’s why you shouldn’t do it alone*,* you know what I mean?*

Throughout this early period, participants like Shawn, a nonbinary person in their 40s, indicated that dealers marketed products to cater to both established modes of use as well as emerging preferences, contributing to the notion that the supply was specifically geared towards certain routes of administration:

*Shawn: So*,* they’re like shooting dope and smoking dope. And you’ve got to let them [dealers] know*,* “I want the shooter dope or the smoker dope.” Because you can’t – the shooting dope doesn’t smoke*,* and of course*,* the smoking dope doesn’t shoot.*

In addition to supply differentiation by intended ROA, participants indicated the smoking transition was also supported by the introduction of successive fentanyl products with rising potency, enabling continued fentanyl smoking, rather than a return to injection use as tolerance increased. ‘Clean’, a type of fentanyl marketed during the summer of 2022 [31], was supplanted by ‘Clean-Clean’ and then ‘Iso,’ which, according to Brendon, a cisgender man in his 30s, was considered the strongest fentanyl product available on the market by the end of 2022:

*Interviewer: Has the quality of fentanyl gone down in your opinion?*

*Brendon: I think it’s gone up. I mean*,* they’ve slowly been introducing stronger and stronger stuff. […] Also*,* I’ve heard of these other substances making an appearance*,* like carfentanil is around*,* and there’s something called Iso something or other. People just call it Iso. And that’s around*,* and that’s been killing people or close to it*,* overdosing people.*

Fears of overdose were a primary motivator of the smoking transition, with participants strongly believing that fentanyl was safer to smoke than inject, although the abrupt and unexpected transition of the drug supply and subsequent changes in mode of use caught some by surprise:

*Shawn: …next thing you know*,* the streets is flooded with it [fentanyl]. And before you know it*,* I’m smoking it. Now*,* I don’t know how that came about. But I know the reason why. I smoke it because I’m mostly alone when I do drugs. And like*,* it’s so easy to fucking kill yourself with fentanyl*,* I just decided to smoke it.*

*Interviewer: And smoking feels safer?*

*Shawn: Smoking is a lot safer.*

While this sentiment was not universal, many supported this claim by referring to extensive overdose experience when injecting fentanyl in contrast to smoking. However, as Lana mentioned previously, smoking is not a guaranteed route to avoid overdose.

### Individual transitions

**Fig. 1:**
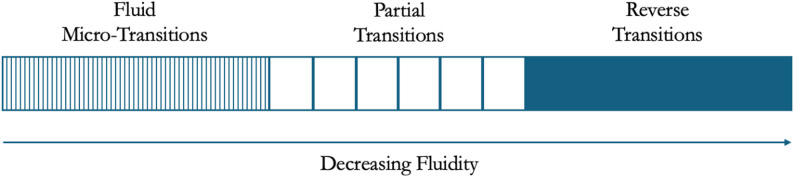
Spectrum of mode of use transitions

We initially identified three broad categories in terms of changes in opioid consumption patterns: (1) injecting fentanyl to smoking fentanyl; (2) injecting other opioids (primarily heroin) to smoking fentanyl; and (3) smoking fentanyl to injecting fentanyl. Additionally, we found a fourth group whose current mode of use was fluid, involving both smoking *and* injecting fentanyl. However, when we examined these use patterns more closely, what emerged resembled a spectrum of transition graduated along degrees of stability over time (see Fig. 1). We therefore re-conceptualized the idea of an ROA transition to include these gradations. At one extreme were those whose daily or weekly use was multidirectional and multimodal, what we term here ‘fluid micro-transitions’; in the middle of the spectrum were those who had mostly shifted from one primary ROA to another while infrequently incorporating other ROAs situationally; and at the opposite extreme were those who had switched entirely in accord with reverse transition patterns from one mode to another, reporting no deviation. These differing transition types express both a directionality of behavioral change and how this change endures over time, from a reportedly complete and consistent switch to frequent movements back and forth.

### A) Fluid micro-transitions

Particularly compelling were reports from participants of how they combined multiple modes of fentanyl administration on a frequent basis. They described a landscape where fentanyl ROA was adaptable by situation, allowing for experiential variety and responsiveness to different needs—beyond using non-injection routes to test for potency or gauge overdose potential [40]. For those who continued to both smoke and inject, this new modality of smoking fentanyl provided previously absent opportunities for choice, refined by social context; desired somatic effect, including from polydrug use; and influenced by considerations of economy and venous access.

### I) Social context

Some people shared that their decision whether to smoke or inject at a given time and place depended on awareness of the feelings of others. This included sensitivity to both peers using drugs and members of the public while emphasizing the ease of smoking relative to injection when preferring to avoid attention. Valerie, a cisgender woman in her 40s, thought social groupings were particularly determinative of her use:

*Interviewer: How do you like to use the clean? Do you smoke it*,* or inject it*,* or both?*

*Valerie: All the above. I put it on foil. I put it in the bong. I inject it. I muscle it. It just depends on what group I’m with. You know what I mean? If I’m with a shooting group*,* then*,* of course.*

The realities of street-based use mean that people using different drugs and modes of administration often interact and share social space. This is especially true in a densely populated neighborhood such as San Francisco’s Tenderloin, a socioeconomically deprived downtown area where different types of drugs can be procured. Many, including paradoxically some people who themselves inject, are afraid of or uncomfortable with syringes and find witnessing injection to be painful or upsetting. Some we spoke to, including Winston, a cisgender man in his 20s, choose not to inject in front of people who are uncomfortable with it and will smoke instead when they are present:

*Interviewer: Does how you use change when you’re with other people versus alone?*

*Winston: Sometimes*,* yeah. If people are uncomfortable with shooting*,* I won’t do it*,* I’ll respect that and I won’t do it in front of them because I understand. It’s not something very pleasing to look at and to see. So*,* if someone’s not comfortable with that*,* then I respect that and I won’t do it. I’ll just smoke it.*

In addition to its social nature, smoking has fewer onerous preparation requirements, allowing for greater flexibility in use, opening additional venues for consumption. As Jamie, a cisgender woman in her 30s stated, *“…the whole*,* looking for it [black tar heroin]*,* trying to get it*,* shooting it*,* trying to find a vein*,* all that little cycle of mayhem…”* dynamic of injection use had been supplanted by an ROA that was simpler, even when using one of the more elaborate smoking devices. Participants reported that during their injection days, they would often crouch in unsanitary and unsafe locations, rush through their process and try to move along as soon as possible. Ashtyn, a cisgender woman in her 40s who described eventually returning to injection (see below), reflected on the advantages of dabbing—where the tip of a heated metal or ceramic tube is gently placed on the fentanyl, with the resulting vapors inhaled—when living homeless, explaining that smoking was more feasible in a public place compared to injecting, both in terms of practicalities and her preference to hide her use:

*Ashtyn: You know what*,* the dabbing seemed to last longer [than injecting]. But that’s only because I could just like take a dab here and there all the time*,* you know? It was that convenient and easy to just heat up a rod and take a hit and then put it away*,* whereas like shooting up was a whole production. I had to get water and a cotton. And I had to be hidden because I’m not going to do it in front of people where you could take a dab almost anywhere if you were quick and efficient about it*,* you know?*

Participants were aware of the publics’ perceptions of drug use in the Tenderloin and were particularly observant of their surroundings, often attempting to shield passersby—especially children—from witnessing their use, despite the open and social nature of smoking drugs.

### II) Desired effect

Although smoking fentanyl allowed people previously injecting it or other opioids to achieve an equivalent high, particularly when using bongs or dabbers, some who smoked and injected explained the continuing draw of injection. Bri, a person in their 20s, described the immediacy of injecting as an attraction:

*Interviewer: Why would you choose one or the other? Why smoke it? Why inject it?*

*Bri: Well*,* they both have different effects in different ways. When you inject it*,* it’s like straight into your bloodstream*,* straight to your brain. So*,* when it goes straight to your brain*,* you know*,* you’re going to feel it right away. When you’re smoking it*,* it’s got about a 15-minute*,* seven-minute interval from it going from your lungs to your bloodstream to your brain. So*,* that’s completely different. It’s*,* like*,* eased into your system compared to just [snaps fingers] put right in there.*

Some, like Emma, a transgender woman in her 30s, reported they could be caught unaware by the delayed onset of smoked fentanyl described by Bri: *“…even smoking it*,* it comes on pretty fast. But also it’s the initial hit*,* and then checking in a few minutes later and it’s kind of settling in. And I don’t think people realize how high you are from the hit until you actually take some time to feel it.”* Others said injected fentanyl remained an established pattern of use for them, especially among those seeking a strong initial rush but its use was largely contingent upon venous access. Kian, a cisgender man in his 30s, explained his rationale and methods:

*Interviewer: […] When you do fentanyl*,* do you smoke it*,* inject it*,* or some other way?*

*Kian: It’s a tossup. I either – partial*,* part of the time I smoke it*,* part of the time I inject it. […]*

*There’s an equal*,* you know*,* pro/con to each one of them. I prefer not to have to use needles sometimes*,* but I also prefer that punch or that boo-yah that you get from injecting. So*,* you know*,* it’s really on the day or the hour*,* like*,* how I’m feeling. If I – Sometimes I catch myself doing this a lot [rubbing arms] feeling for veins or anywhere to inject. And if that’s easy to find then I’m going to inject. […] And if it’s not easy to find or if it’s cold and I’ve got*,* like*,* goose pimples*,* then I’m going to smoke it.*

### III) Economic considerations

Economic concerns also could influence micro-transitions. Valerie sometimes desired the fast onset of injected fentanyl, like Bri, but her choice was influenced by her need for injection assistance which could conflict with her management of withdrawal symptoms. She needed to find a ‘street doctor’, a fellow PWUD considered expert in their ability to inject someone else, but whose assistance came with additional costs that needed to be balanced against prioritizing drug effect:

*Interviewer: And when do you choose to inject versus when do you choose to smoke?*

*Valerie: Well*,* injecting*,* I would do it if I got to be high now*,* I can’t wait for everything to kick in… […] And then*,* plus*,* too*,* it’s hard to find [street] doctors now… ’Cause what people do is*,* they’ll look at what [drugs] you have*,* and they’ll go*,* “I want that pink piece*,* right there*,* plus $5*,*” but just that little piece might be $30 worth… $30 worth of fentanyl over a shot. No. […] [But] you got to give them something. You got to pay the piper. You know*,* ‘cause finding a doctor and a good doctor*,* is hard ‘cause you don’t want to be stuck 20*,*000 times. You don’t want to be stuck ‘cause [of] abscesses and all that other stuff*,* too. So*,* you want to be stuck one time and one time only…*.

Randall, a white cisgender man in his 30s who injected himself, found injection more cost-effective than smoking, and believed injecting a tenth of a gram of fentanyl was comparable to smoking an entire gram:

*Interviewer: […] It sounded like you were saying that the cost made a difference as to whether you inject or smoke as well.*

*Randall: Mm-hmm […] because then I’ve only got to do a miniscule amount compared to smoking […] Say you only have to do a point [0.1 g] on an injection instead of smoking like a gram to get the same feeling.*

For those whose veins were still accessible, being able to rely on two ROAs provided greater flexibility to prevent opioid withdrawal and ensured that financial resources spent on drugs provided sufficient impact.

### IV) Polysubstance use

While many participants engaged in polysubstance smoking, some who had largely given up injecting, found the attraction of injected polydrug use remained strong. Although Karl, a white cisgender man in his 30s, aspired to switching to smoking entirely, he made an exception for injecting cocaine and fentanyl together:

*Interviewer: All right, so how do you use your fentanyl?*

*Karl: Smoke it and sometimes*,* IV. But prefer to smoke. Trying to stop shooting up*,* really.*

*Interviewer: Why do you prefer smoking?*

*Karl: Because I have like really bad veins. So*,* you can kind of achieve what you’re trying to do by smoking*,* without having to use a needle. But for a shorter period of time*,* I’d say. […] I might try to make a transition permanently*,* but it’s not easy. I think the coke is the biggest factor of that. […] No smokable high can achieve the rush from a speedball.*

Oliver, a transgender woman in their 30s, who had transitioned to smoking fentanyl after losing many friends to overdoses, continued to inject when using fentanyl in combination with methamphetamine in a cocktail they called a ‘Bruce Jenner’ (referencing the athlete who had transitioned from male to female, now known as Caitlyn Jenner), or alternating between modes of use at one time to achieve the desired balance:

*Interviewer: Yeah. Okay, and do you ever inject and smoke at the same time?*

*Oliver: Yes*,* sometimes. I’ll smoke fentanyl*,* and – I’ll shoot some meth and then*,* smoke some fentanyl. That’s the thing that I have done. I prefer to do it like that. I’ll take about a tenth of methamphetamines and then*,* inject that. And then*,* smoke about the same amount you just seen right there. And it balances it out. Or I’ll take that right there and then*,* mix it and do a ‘Bruce Jenner’. […]*

*Or it’s even I’ve injected some fentanyl and then*,* been like*,* “Oh*,* I need to smoke some methamphetamines to speed myself up.”*

The versatility of fentanyl ROAs allowed its consumers to make finely tuned adaptations to their consumption, whether between each dose or over longer periods, tailoring these to their budget, social situation and desired effects from one or more substances.

### B) Partial transitions

Similar to the individuals classified as fluid micro-transitioners, a subset of participants indicated that although they incorporated both injection and smoking into their use repertoires, they were more likely to use through only one ROA, transitioning between ROAs with far less frequency than the fluid group. For some, this meant injecting more frequently, while others used more often by smoking. Partial transitioners portrayed fentanyl smoking as a more casual means of using the drug, both in terms of its effects and in contrast to the more involved process of injection, notably contributing to their transition. Jamie described her own transition from injecting heroin to smoking fentanyl in positive terms, giving her a greater degree of control over the challenges of managing her addiction and participating in everyday life:

*Jamie: And it’s actually a less consuming high. I don’t know if that’s a good way of putting it. But*,* I switched from using black tar heroin over a year ago now*,* maybe a year and a half ago*,* and I was shooting tar. And*,* you know*,* just the whole*,* looking for it*,* trying to get it*,* shooting it*,* trying to find a vein*,* all that little cycle of mayhem would consume my whole day*,* you know*,* to the point of it was just like Groundhog’s Day—it was horrible. And*,* you know*,* with fentanyl it’s like I can smoke some and then I can*,* like*,* have a day. I can go and I can get a job. I feel so much more*,* like*,* active as a member of society.*

However, she also appreciated the option of an intense fentanyl effect when she chose, without the side effects of injection. *“Like*,* I enjoy the fact that I can get kind of as high as I want to and only need to smoke versus*,* like*,* having to*,* like*,* shoot up and*,* like*,* you know*,* ruin my veins and everything else that comes along with*,* you know*,* heroin use.”* Despite these advantages, she continued to sometimes inject methamphetamine and fentanyl together, depending on the situation:

*Interviewer: […] has the cost changed the way that you use your drugs*,* like smoking or injecting […] how it’s so inexpensive?*

*Jamie: It doesn’t change*,* like*,* the injection or non-injection aspect of it. Like*,* that’s*,* like*,* kind of*,* like*,* a forgone conclusion. Like*,* it’s either going to be shot or it’s not going to be shot*,* and it has nothing to do with the price. It just has more to do with situational and*,* you know*,* I guess*,* like*,* cultural almost*,* like*,* than anything else.*

Aiden, a cisgender man in his 50s, also followed a partial transition pattern but on a less common trajectory. Aiden and his wife had recently arrived in San Francisco from St. Louis, Missouri where he injected fentanyl and methamphetamine, using fentanyl to relieve the pain from a shattered ankle. On arriving in San Francisco, he tried to smoke it but found that while it provided relief from opioid withdrawal symptoms, it was not effective for pain management, nor did it provide a pleasurable high. He attributed this to a potency disparity between the fentanyl sold in the two cities, leading him to prioritize injection use while also acknowledging that smoking could still be useful for relieving withdrawal symptoms:

*Aiden: […] we never smoked it ourselves until we were here. And like I said*,* neither of us see the point behind it*,* you know. It will make you not sick*,* but you seem to go through way more*,* and you don’t get the same effect. Now*,* like I said*,* for me*,* a lot of times*,* especially with my ankle*,* I want the quicker effect […]*.

*Interviewer: Yeah. So*,* like*,* the effect that you’re looking for that smoking doesn’t give you*,* what is that? So*,* what is the effect of shooting it?*

*Aiden: Okay. It doesn’t seem to help with the pain when I smoke it. If you’re going for a euphoric feeling*,* I don’t get none of that. Where shooting it*,* instantly*,* instant pain relief*,* instant not sick*,* and smoking it*,* that’s all it seems to do*,* is make me not sick. I don’t get no other real feeling out of smoking it.*

In contrast with others in this study, Aiden did not have extensive vein damage from injecting black tar heroin, thus not precluding a return to injection use. As well as effectiveness and speed of onset, value for money were also part of Aiden’s calculus:

*Interviewer: Yeah. When you’re using fentanyl*,* what effects are you looking for mainly? Like*,* are you looking for*,* like*,* relief from withdrawal*,* pain relief*,* euphoria?*

*Aiden: Pain relief and withdrawal mainly. We do pretty good with maintenance where a lot of people don’t think you can manage that. And then there’s the occasional time you just feel like getting messed up*,* and then the euphoric effects comes*,* you know*,* which for us isn’t that often*,* but part of that*,* too*,* is the quality*,* you know. You have to do so much. We couldn’t afford to.*

However, in addition to this economic consideration, Aiden would also smoke fentanyl in social situations where it was offered to him freely, which he acknowledged was common: *“You know*,* if it wasn’t for other people sharing or*,* you know*,* like*,* people we’ve been around smoking it*,* after the first couple times*,* we wouldn’t even smoke it*,* again*,* you know.”*

### C) Reverse transitions

 In line with reverse transitions identified in research among people injecting heroin, some participants indicated that they had made a stable and durable transition from injection use to smoking without returning to injection use for any of the reasons advanced by the other groups. Gena, a person in their 40s, had injected methamphetamine and heroin but was able to give up all injecting when they started smoking fentanyl. Their description, however, suggests an ambivalence as to whether the adoption of fentanyl, despite its advantages in stopping injection, was ultimately beneficial:

*Interviewer: Yeah. So do you still do that ever?*

*Gena: No*,* it’s actually been three years off the needle.*

*Interviewer: Okay.*

*Gena: And fentanyl is kind of the fake hero in that*,* you know*,* because I was looking for a way out of the shooting IV drug scene*,* and it kind of just took over – the fentanyl smoke.*

The notion of fentanyl as ‘fake hero’ was encountered among several who had reverse transitioned—*“It [fentanyl] got everybody off the needle. It did do that.”* (Lana)—suggesting that while they valued its introduction for facilitating their transition from injection use, they found that it introduced new dynamics in their substance use. Gena and Lana both used methamphetamine somewhat infrequently in the past, when injecting was their primary ROA, however the transition to smoking fentanyl rendered methamphetamine essential. For Gena, smoking methamphetamine in combination with fentanyl helped reduce her fentanyl use overall:

*Gena: And with the crystal*,* it’s to stretch out the fentanyl*,* especially if you’re smoking on foil*,* because [you can] budget and stretch out your usage so you’re not just smoking*,* smoking*,* and then going back to get some more… It’s like a four to one ratio of fentanyl to meth*,* more fentanyl and just a little bit of meth to melt in there*,* because meth can overpower the fentanyl.*

Lana, however, felt that fentanyl’s emergence and supplanting of heroin limited her freedom, causing her to feel as if she had to use methamphetamine as an overdose prevention mechanism:

*Lana: Fetty just took over every – I can’t leave the Tenderloin. I’m chained to this place because you can’t find fentanyl nowhere. […] [But with] fentanyl*,* you’re out [overdosed] like that. You’re fucking just out. I got high out there [Tenderloin Center consumption space] because I didn’t want to be sick in here*,* but I had to fucking bong freaking crank [methamphetamine]. Otherwise*,* I would be fucking out.*

Others, like Clay, a cisgender man in his 30s, saw their transitions as an explicitly harm reduction approach to their opioid consumption. Before he moved to San Francisco from the East Coast Clay had been injecting heroin, and fentanyl when heroin was unavailable. He initially avoided fully switching to fentanyl for fear of overdose but transitioned to fentanyl smoking after finding that San Francisco’s fentanyl was less potent than in the East, describing this transition as a complete and long-term change:

*Clay: I don’t inject at all just because I kind of came to the decision that if I was doing any sort of drugs*,* if I had to hurt myself*,* literally hurt myself like punch a hole in my skin any time I was using a drug*,* then it was definitely a negative thing in my life. I couldn’t really make account for that*,* you know what I mean?*

*Interviewer: So, you’re not injecting anymore?*

*Clay: No. It’s been a hot minute since I have even tried to do a shot.*

For those switching from injecting opioids of lower potency to smoking fentanyl, the potential to titrate the dose over time according to the body’s response was particularly important for overdose prevention. Like Clay and Gena, some took this opportunity to stop all drug injection. Eric, a cisgender man in his 50s, had previously injected heroin and methamphetamine but switched completely to smoking fentanyl without ever injecting it:

*Eric: (Fentanyl) kind of saved me because I – I didn’t shoot anymore. So that’s one thing I do credit fentanyl with*,* it took the needle out of the game. And that’s cool. […] I never shot – I – I don’t shoot that. That’s just a little much.*

### D) Return to injection?

While we primarily encountered people who had moved from injecting to smoking fentanyl, transitions from a period of smoking to injecting fentanyl were rare but not unheard of, as in the case of Aiden above, who adopted smoking upon arriving in San Francisco but returned to prioritizing injection use. Ashtyn’s transition to exclusively smoking fentanyl from injecting, which lasted for a sustained period, reversed after the loss of her smoking equipment, emphasizing the fragility of this transition for some. Her dealer had encouraged her to take up dabbing: *“And so I threw all my needles away*,* and me and him dabbed the fentanyl together. And it actually worked for me and I got high*,* and so I actually was clean off the needle for almost four months.”* However, the theft of her smoking equipment compelled her, with regret, to return to injecting:

*Ashtyn: And then I moved here to San Francisco with all my stuff to do it that way*,* and my bags were stolen the first week I was here because I was homeless and I couldn’t stay awake anymore*,* you know what I mean? […] And so I didn’t have the money to buy new equipment*,* the expensive torches and the dabbing tools*,* so I went back to shooting. … Yeah*,* I had quit shooting*,* which is a really big accomplishment for me. It was a huge*,* big deal. I think I overall felt completely better not shooting up*,* period*,* because it’s got to be hard on your body putting things right into your bloodstream*,* you know what I’m saying?*

While the variety of means, equipment and techniques associated with fentanyl smoking afforded participants the possibility for more varied experiences than injection, early adoption of more intense smoking forms may preclude uptake of other means of smoking. Rather than adopt an alternative smoking method, with means and techniques on ample display at the Tenderloin Center, Ashtyn found returning to injection was both more achievable and in line with what she sought from her fentanyl use.

## Discussion

Fentanyl’s versatile ROAs have allowed San Franciscan PWUDs to fashion their own styles of consumption that differ from historical patterns of ROA transitions. The drug’s potency and the marketing of smokeable fentanyl products of reportedly increasing strength have facilitated wide scale transitions in ROA. Patterns of change in mode of use fell on a spectrum, with fluid multimodal micro-transitions at one extreme and stable reverse transitions at the other.

On the reverse transition end of the spectrum, some have seized the opportunity to extinguish injecting entirely from their repertoires, referencing the resulting health benefits and improvements to their overall functioning, while others were compelled by venous access issues. Meanwhile, at the opposite end, consumers were selecting their modes of use flexibly, according to multiple considerations. In the middle were those who had largely transitioned to a new dominant mode but occasionally switched back to former methods. Given the constrained circumstances in which many of the participants lived, hemmed in by poverty, housing problems, participation in an unregulated drug market and the demands of opioid and other dependencies, the degrees of flexibility they fashioned in their fentanyl use allowed greater self-determination. This also revealed an ability to adapt according to constantly changing circumstances.

The question of whether ROA transitions will endure over the long term is an important one. Several of those who had given up injecting, like Clay and Gena, were confident that this was a long-term change. Others, like Karl, were struggling to give up injecting completely in favor of smoking. The case of Ashtyn also highlighted the importance of the availability of affordable smoking equipment in maintaining the transition. Some equipment, such as dabbers and torches, are too expensive to be provided free by most harm reduction services but foil and glass pipes are more affordable. The role of harm reduction services, largely originating in the distribution of sterile syringes in the wake of HIV, is likely to be key in supporting and sustaining ROA transitions [41] but disparities in access to these programs [42, 43] amidst an increasingly anti-harm reduction federal posture may negatively affect their outreach potential. Recent research has highlighted how integration of additional services at existing harm reduction service providers, including drug checking [44] as well as PrEP and buprenorphine initiation [45] can reach more diverse populations. Integration of related services, e.g., infectious disease testing, wound care, or other types of services, such as aesthetician hours for hair and skin care or food programs, may additionally help to reach minority populations who snort or smoke more commonly than inject [46]. An analysis of US treatment admissions has found that administration of opioids by smoking or nasal insufflation as opposed to injection has been on the rise since 2000, with strong regional variation [32]. We did not interview any individuals in San Francisco who included nasal insufflation as a preferred ROA, but this mode is more common on the US East Coast [32]. The distribution of smoking devices has encountered political opposition at the federal [47] and local level [48] that could bring into question the durability of ROA transitions and their uptake in other US locations.

More broadly, our findings on ROA transitions and their connection to the social contexts, motivations, and needs of PWUO should inform harm reduction and related interventions. Building interventions with these findings in mind can help ensure that they are responsive to the lived experiences of people who use drugs and their needs based on varied patterns of use. Several factors explored in this paper and related research problematize the health impacts of ROA transitions. While most participants had not identified rising opioid tolerance as a deterrent from continued smoking, others reported seeking out the perceived highest potency fentanyl product available, eschewing less potent options [31], although other research with this study population found, contra earlier research on heroin use [49], that potency was not the most salient factor driving use patterns [37]. Several participants who had used fentanyl in other locations emphasized that San Francisco fentanyl was relatively weak in comparison, but drug purity and potency are highly variable and are mediated by opioid tolerance, health and ROA. Vicissitudes in heroin purity have been shown to influence overdose [50] and fentanyl purity variance is likely similar or even more dangerous in terms of overdose rates.

The popularity of smoking devices that can mimic the bolus effect of injection use, e.g., bongs and dab rigs adapted from cannabis use [31], suggest many PWUDs continue to value a combination of potency and efficiency in drug administration that, alongside rising tolerance, may influence use patterns and overdose risk. Additionally, the finding that partially smoked fentanyl residue remains bioavailable, is easily shared and can be difficult to differentiate from other drug residue, e.g., methamphetamine [31, 37], may create new pathways for unintentional overdose. While evidence to date supports a reduced overdose risk from fentanyl smoking compared to injecting [51, 52], the longer term pulmonary and circulatory effects are as yet unknown, and may be further complicated by the spreading presence of xylazine in the fentanyl supply [53–55]. In the current health emergency, the immediate need to intervene to prevent sudden deaths is paramount but a careful eye should be kept on other consequences.

This study has several limitations. These findings were generated from qualitative research that used purposive, nonrandom sampling. Qualitative research is useful for hypothesis generation but is not a satisfactory method for determining causality. While recruitment occurred at several locations in San Francisco, these findings may not be representative of how people use fentanyl or other opioids in other parts of the city or other cities in the Bay Area. Much of the sample was white, which may not accurately represent the drug using population in San Francisco. Findings may not be comparable to other US locations due to the unique characteristics of the city’s urbanicity, drug market and network of service agencies. However, the tight focus on ROA transitions in San Francisco illuminated broad transition patterns, potentially serving as a test case for other municipalities. This study used rapid ethnographic assessment techniques and there was no long-term follow up with participants to determine whether their transitions had endured over the study period. Participants were not eligible to be interviewed multiple times although some were encountered during both recruitment weeks. This limitation was partially addressed with brief conversations checking in with prior participants and through observations of drug consumption practices in the consumption space at the Tenderloin Center or on the street when feasible. Social desirability bias may have influenced some responses, but data triangulation between interviews, observations and fieldnotes was intended to mitigate this bias.

While historical patterns of ROA transitions among people using opioids have been conditioned by rising tolerance to their euphoric and analgesic effects, fentanyl use in San Francisco has demonstrated a striking break with this history that has potential implications nationwide given rising use of fentanyl by smoking or snorting. The stability of these transitions, their unknown long-term health implications and influence on overdose risk bears scrutiny, particularly amidst an increasingly adulterated drug supply.

## Data Availability

Due to the sensitive nature of the research—e. g. potential legal action against participants for sharing information about illegal activity, and harm to members of the research team for divulging such information—the University of California, San Francisco Human Research Protection Program (IRB) has limited access to this data only to approved study personnel. While the transcripts are de-identified, participants did not consent to making their data available to those outside of the research team and the detailed experiences shared may plausibly allow for participant identification. Researchers who meet the criteria for access to confidential data may send requests for consideration to the Human Research Protection Program (IRB) at the University of California, San Francisco at 415-476-1814 or IRB@ucsf.edu.
